# Responsibility and disrespect: reply to Tsiakiri

**DOI:** 10.1007/s11019-026-10344-4

**Published:** 2026-03-18

**Authors:** Adam Ehlert, Joar Björk

**Affiliations:** 1 Centre for Research Ethics and Bioethics, Uppsala University, Uppsala, Sweden; 2 Swedish National Centre for Priorities in Health, Linköping University, Linköping, Sweden; 3 Department of Research and Development , Region Kronoberg, Växjö, Sweden

In a recent paper, Tsiakiri (2025) defends the use of luck-egalitarianism (LE) in health-care priority setting against the charge of *disrespect*. She claims that a ”responsibility-sensitive healthcare system”, that is, a healthcare system that relies upon or is in accord with certain luck-egalitarian principles regarding priority setting decisions, can be compatible with respect for patients. She supports this claim by alluding to the Doctrine of Double Effect and Kant’s Formula of Humanity. The argument is further underpinned by the idea that people have a perfect duty (towards themselves and/or others) to promote their health in certain ways, which includes refraining from certain health-damaging activities.

In this reply, we criticize Tsiakiri’s argument on several grounds. First, we attempt to show that the analogies used, involving the trolley problem and the case of suicide, fail to do the normative work that they would need to do. Second, we argue that Tsiakiri’s Kantian framework presupposes certain ideas of agency and control that are incompatible with the empirical reality of health behavior. Based on this, we conclude that Tsiakiri fails to show that a responsibility-based system is compatible with respect. Third, we argue that even if people have duties such as those suggested by Tsiakiri, it does not follow that the health care system ought to enforce them. Even if it could be shown that a responsibility-based is compatible with respect, this does not mean that such a system should be actualized.

## Summary of Tsiakiri’s paper

Tsiakiri begins her paper by explaining the increasing interest in the use of LE in health care. Non-communicable diseases are becoming increasingly common (Lim et al. 2013), and ideas regarding the importance of responsibility has been shown to play a role in the public’s opinions of healthcare (Salkeld et al. 2007). She introduces the responsibility-based priority setting principle based on LE, which, very briefly, will give lower priority to patients who are responsible for their conditions, all else being equal. She discusses some objections to such principles, restricting her focus to what she calls “respect-based objections”. She never clearly defines (dis)respect, but instead uses it as an umbrella term for a set of different concerns which are frequently voiced regarding LE and responsibility – namely, harshness (Anderson 1999), intrusiveness (Wolff 1998), and discrimination (Björk 2021). She proceeds to introduce her arguments for why LE and responsibility-based principles can avoid these charges, arguing that there are valid respect-based reasons to take responsibility into account.

We will now focus on those aspects of the argumentation that we find most challenging.

## The doctrine of double effect and the trolley dilemma

Tsiakiri reinterprets the Doctrine of Double Effect (DED) and uses the trolley problem to illustrate her point. She starts by presenting the classic trolley case (Foot 1967), which involves five workers tied to one branch of a railroad track, one worker tied to the other branch, and an outsider with the capacity to operate a switch to divert the trolley to either branch. She then asks readers to imagine a 1-to-1 case, where there is a *prudent* worker on one branch and an *imprudent* worker on the other branch. Since one of them is responsible for their predicament and not the other, Tsiakiri claims that there is a clear solution to this version of the trolley problem: save the imprudent worker. She makes further changes to the thought experiment so that instead of workers on a railroad track, the individuals involved are two people in need of a cornea transplant, where one is responsible for his poor eye health while the other is not. Tsiakiri maintains that the reasoning ought to be the same in this case: we should give the cornea transplant to the person who is not responsible for her poor eye health.

However, we find the argument misleading. In its classic conception, the DED is predicated on the (assumed) morally relevant difference between *intended* as opposed to merely *foreseen* harm. Indeed, this is what is meant by “double” effect. If the only relevant form of harm in a case was of the merely foreseen kind, there could be no talk of potential “double effects”. It is assumed by defenders of the DED that intended harm is, *ceteris paribus*, worse than merely foreseen harm. Compare a terrorist bombing with a tactical bombing of a military target. In both cases, there is excessive harm. However, in the terrorist bombing the harm is intentional, whereas in the tactical bombing the harm is (supposedly) a merely foreseen harm. Otherwise put: if there were means to reduce the harm, the decision-makers in the tactical bombing situation might be expected to utilize these means, whereas the terrorist would not be expected to do so. This difference, so most would agree, provides one argument why terrorist bombings might be considered ethically worse than tactical bombings. Returning to the issue at stake in Tsiakiri’s argument, we argue that LE is a theory which argues for harm of the *intended* kind. Tsiakiri paints a picture of an imprudent patient being down-prioritized for some kind of treatment in comparison to a prudent patient, to the medical detriment of the imprudent patient. This can reasonably be seen as “harming” the imprudent patient, as they are made worse off than they otherwise would have been^1^. (True, there may be alternative conceptualizations of harming, according to which the imprudent patient is *not* harmed, but taking that argumentative route would leave DED out of the picture as DED *ipso facto* concerns situations involving potential harm. So, for DED to be relevant in this discussion we must accept that the priority setting situation actually does involve potential harm.) Crucially, this harm is intended, not merely foreseen. Responsibility-sensitive healthcare does not *foresee* the disadvantage imposed on the imprudent, but rather relies on this very disadvantage as part of its normative justification. LE argues that the imprudent *must* bear the consequences of their actions (to prevent injustice to the prudent, to achieve incentive effects, etc.). Hence, it is not at all clear that LE would reduce the harm to the imprudent, even if possible. Somewhat harshly, LE is in this sense more like a terrorist bombing than a tactical strike. Summing up our argument, we reiterate that LE deals with *intended* harm and therefore the appeal to DED fails, and the analogy loses its bite. To save this part of her argument, Tsiakiri would need to demonstrate that incentive effects are merely *foreseen* rather than *intended* which, we expect, she cannot consistently do. Hence, the DED analogy cannot ground the claim that LE is not disrespectful.

The proponent of LE is likely to reply, here, that their main objective is different and does not entail harm at all. They could claim, for instance, that letting people bear the costs of their past choices is not harming them but instead respecting their agency and treating them with the respect that they deserve. We respond that they are *also* trying to incentivize people to act more responsibly by letting them bear these costs. Indeed, the intention to achieve incentive effects is explicit in many texts on responsibility-sensitive healthcare policies system (Smart 1994; Daniels 2011; Miraldo et al. 2014; Schmidt 2015; Andersen and Nielsen 2016; Davies 2021). Notably, this feature of LE operates *– by necessity –* through intended harm. Indeed, Tsiakiri is committed to accepting precisely this, since she says that the policies do their work by “disapproving, demoralizing and even stigmatizing people who engage in unhealthy actions, behaviors and habits” (Tsiakiri 2025, p. 320). In fact, we suspect there may be several layers of (potential) harm at play here. First is the harm to imprudent patient themselves, supposedly to incentivize them to change their behavior. But Tsiakiri goes on to state that LE policy “nudges the general population into the adoption of a healthier lifestyle” (ibid.). This, then, is using the fate of imprudent patients to set an example for others, which we consider a case of using intended harm on one group of people to benefit another group, i.e. using some people as a means for the benefit of others.

## The case of suicide

Tsiakiri further makes an analogy between suicide and overeating, which we think is very illuminating – but for entirely different reasons than Tsiakiri herself.

To start off, we note that healthcare professionals do not commonly simply shrug their shoulders at suicide attempts – rather, they try to save the lives of suicidees if possible. One reason for this is the assumption that suicide attempts rarely stem from a rationally deliberated wish to die. Is Tsiakiri ready to challenge this policy by stating that most suicides are rational, and that suicide attempts should not receive treatment?^2^ This is the bullet she would need to bite to save the point of the suicide analogy – but we expect she would not like to bite it. In any case, we propose that just like suicides cannot generally be contributed to a well-considered wish to die, neither can overeating be contributed to such a wish. Indeed, we believe that this is to seriously misrepresent the motive, and hence the moral meaning, of overeating. Neither overeating nor smoking should be understood as purely self-destructive behavior in Kant’s sense. Indeed, we assume that most people who overeat would prefer to overeat *and* live – as irrational as this may be. The human irrationality at play here ushers in our next point: both Kantianism and LE overstate the occurrence and/or^3^ normative relevance of rational self-governance when it comes to life-style “choices”. Empirical studies indicate that health behavior is heavily influenced by what Kant would see as “heteronomous” factors (Shelton and Balint 1997; Martens 2001; Marmot 2009; Temkin 2011; Marmot and Bell 2016; Pillutla et al. 2018; World Health Organization 2021).

In reply, luck egalitarians could state that they do not, in fact, claim that decisions such as committing suicide or overeating are *fully* rational, or that people are *fully* responsible for such decisions. It would be enough for LE proponents to say that as long as people are *partly* responsible for these and other decisions, this is enough to deprioritize them. But consider what that would mean in the imagined scenario of two patients, A and B. A is a health fanatic who thinks about their health all the time, exercises seven days a week and eats only the healthiest food. B is also health-minded, exercises most days of the week and eats mainly healthy food. Once or twice, however, B takes a rest day and indulges in some wine or chocolate. Despite their good behavior, both A and B turn out to need a new kidney. A has done absolutely nothing risky, whereas B’s occasional lapses may have increased their risk for kidney failure at least slightly. Hence, B is *partly* responsible.^4^

If Tsiakiri wants to claim that B has violated a perfect duty towards themself (i.e. the duty to preserve one’s health), and therefore should be deprioritized for a kidney transplant, this seems overdemanding, to put it mildly. But if they want to deny this, they are committed to explaining what should count as the “threshold level” for preserving one’s health. Unless this is done, it will be impossible to apply the principle to real cases. However, we think it would be difficult – in either LE or Kantian terminology – to provide a satisfactory account of such a threshold feature in the theory.

There is, however, yet another option available for Tsiakiri. She can assert that imprudence should only ever be used as a tie-breaker and therefore only become operational in *ceteris paribus* cases, i.e. when two patients are exactly alike in all other relevant aspects. (To assess this, a number of factors relevant for priority setting such as health need, outcome of treatment, cost-effectiveness, time needed to treat, etc., could be investigated until it was established that the patients differed *only* with regards to prudence.) However, we do not think this is a viable strategy. If one wants to designate one aspect (here: responsibility) the status of a tie-breaker, one needs to explain why it should not be given more relevance than that enjoyed by a tie-breaker. We find it paradoxical to use responsibility in this particular way, as it entails on the one hand claiming that it is of minor importance, and on the other hand granting it big impact over policy (cf. Jølstad and Gustavsson 2023).

## The empirical/normative divide

On page 317, Tsiakiri consciously separates normative from empirical claims in the debate about responsibility. We think this move may be too rash. If *empirical* evidence signals that it may very rarely be possible to prove patients’ imprudence in the clinical setting (as opposed to in laundered theoretical examples), this also provides a *normative* reason not to speak of imprudence in healthcare priority setting (Sisk et al. 2020). Indeed, Kant famously quipped that “ought implies can”. If it is true that it will rarely be possible to prove whether patients have been imprudent, it will also rarely be the case that we ought to treat patients differently based on imprudence (as LE would have it). In light of this, one can question whether policy – as a whole – would really be improved by adding a consideration of potential imprudence. We follow Björk (2021) as we argue that the value of “policy parsimony” may very well outweigh the value of imprudence considerations – *even* to those who care about such considerations. There are several ways to support this claim. First, there is a strong tradition in philosophy to prefer simple theory over complex theory. Philosophers from Aristotle to Aquinas have held simplicity as a virtue and affirmed something like Occam’s Razor, and Kant himself claimed that “principles must not be unneccesarily multiplied” (Kant 1781/1787, p. 538-9). Perhaps more importantly, however, studies in cognitive psychology and decision science show the importance of the speed/accuracy trade-off in human decision-making (Standage et al. 2015). Simply put, there may be situations where the potential added quality by a more complex decision rule does not merit the extra time required to process the rule. Also, overly complex decision-making rules may be so demanding in terms of mental workload that the risk of making flawed decisions actually increases (Byrne 2013). This may be particularly accentuated in those areas of healthcare where there is a lack of time and/or high medical risks (Reale et al. 2023). For these and other reasons, it may be important to keep medical decision-making systems relatively easy (Marewski and Gigerenzer 2012). Furthermore, the human desire to simplify, especially during time pressure, could lead to ethically worse outcomes if, for instance, physicians prefer to *assume* that patients have been imprudent rather than spending time to *establish* this fact.

Furthermore, we think that the empirical/normative separation leaves Tsiakiri’s argumentation unfinished. Even if we would grant her the claim that maintaining one’s health is a perfect duty, both towards oneself and to others, this does not by itself translate to the claim that healthcare should enforce this duty via healthcare rationing. The fact that we have a perfect duty to ϕ does not entail that we should, for instance, use healthcare rationing to enforce people to ϕ. To just cite one example, Kant also argues that it is a perfect duty not to lie, and we could maintain that this is true and still not claim that we should use healthcare rationing to prevent people from lying. The important issue here is not whether there “is” a duty, but whether it warrants coercive institutional enforcement.

The duty not to *steal*, for instance, is a paradigmatic candidate for state enforcement, because breaching this duty entails direct infringement of someone’s rights. Theft is wrongful appropriation of someone else’s stuff. It is reasonable to hold that this creates a claim of justice, warranting institutional enforcement. However, duties such as the duty not to lie are not typically connected to rights violations. It *can* be, like in a case of fraud, but is not always so. The duty not to lie remains a duty of personal morality and does therefore not warrant enforcement. We think that, to buttress her claims, Tsiakiri would need to do more work to explain how we are to understand the (putative) duty to maintain one’s health.

Our own judgment is that a putative duty to preserve one’s health appears more like the duty not to lie than like the duty not to steal. Failing to maintain one’s health (even if morally criticizable) does not ordinarily constitute a violation of some other person’s rights – at least not an *identifiable* person. True, there may be indirect downstream effects on one or many other individuals, but healthcare systems are so complex that it is generally hard to pinpoint exactly which individuals and how^5^. Therefore, treating the failure to preserve one’s health as a ground for rationing would be to extend coercive enforcement far beyond its usual realm of rights violations. Even if we accept a perfect duty to maintain one’s health, this does not by itself explain and justify such a move. Any argument to the opposite would need to explain why, in particular, the real-world context of healthcare is a suitable arena for enforcing the duty.

## Conclusion

Tsiakiri tries to defend the claim that a responsibility-sensitive health care system is not disrespectful. This defense fails on several accounts. First, it uses the example of the trolley problem in a misleading way. Second, it makes use of a faulty analogy between suicide and other behaviors detrimental to health. In addition to this, for reasons of what we call “policy parsimony”, very strong reasons must be provided in favor of responsibility-sensitivity to override its low practical significance. Finally, even if it were the case that individuals have perfect duties to not engage in specific behaviors detrimental to health, it does not follow that the healthcare system should be sensitive to responsibility as a way of enforcing these duties.

## Footnotes

1 On the Counterfactual Comparative Account of Harm, an event E harms P iff P would have been better off if E had not occurred (Klocksiem 2012). On the Causal Account of harm, an event E harms P iff there is a state of affairs D such that (i) E causes D to obtain, and (ii) D is a harm for P (Carlson et al. 2021). On the Negative Influence on Well-being Account, what it is for an event E to harm P is for E to adversely affect P’s well-being (Johansson and Risberg 2022).

2 This might be a tough claim for Tsiakiri to sustain, since many Kantians would claim that suicide is always irrational. For an exception, see Sjöstrand et al. (2013).

3 What we mean by “and/or” here is that it is possible to construe a Kantian/LE position which accepts current evidence on the (non-rational) determinants of health. Such a position would then say something like: “people’s lifestyle ‘choices’ are not really choices, and often a far cry from rational. But the empirical and the normative come apart (as Tsiakiri seems to suggest on 317) and people should simply be punished for not being rational”. This, however, would be yet another bullet we expect most LE proponents would prefer not to bite.

4 We thank an anonymous reviewer for suggesting this example.

5 This is true for real-world priority setting situations rather than “neat and tidy” philosophy-textbook thought experiments.
